# High Altitude Affects Nocturnal Non-linear Heart Rate Variability: PATCH-HA Study

**DOI:** 10.3389/fphys.2018.00390

**Published:** 2018-04-16

**Authors:** Christopher J. Boos, Kyo Bye, Luke Sevier, Josh Bakker-Dyos, David R. Woods, Mark Sullivan, Tom Quinlan, Adrian Mellor

**Affiliations:** ^1^Department of Cardiology, Poole Hospital NHS Foundation Trust, Poole, United Kingdom; ^2^Centre of Postgraduate Medical Research and Education, Bournemouth University, Bournemouth, United Kingdom; ^3^Research Institute for Sport Physical Activity and Leisure, Leeds Beckett University, Leeds, United Kingdom; ^4^The Defence Medical Services, Lichfield, United Kingdom; ^5^Northumbria and Newcastle NHS Trusts, Wansbeck General and Royal Victoria Infirmary, Newcastle upon Tyne, United Kingdom; ^6^Department of Academic Medicine, Newcastle University, Newcastle upon Tyne, United Kingdom; ^7^LumiraDx, Waltham, MA, United States; ^8^James Cook University Hospital, Middlesbrough, United Kingdom

**Keywords:** heart rate variability, high altitude, cardiac patch, acute mountain sickness, non-linear, rating of perceived exertion

## Abstract

**Background:** High altitude (HA) exposure can lead to changes in resting heart rate variability (HRV), which may be linked to acute mountain sickness (AMS) development. Compared with traditional HRV measures, non-linear HRV appears to offer incremental and prognostic data, yet its utility and relationship to AMS have been barely examined at HA. This study sought to examine this relationship at terrestrial HA.

**Methods:** Sixteen healthy British military servicemen were studied at baseline (800 m, first night) and over eight consecutive nights, at a sleeping altitude of up to 3600 m. A disposable cardiac patch monitor was used, to record the nocturnal cardiac inter-beat interval data, over 1 h (0200–0300 h), for offline HRV assessment. Non-linear HRV measures included Sample entropy (SampEn), the short (α1, 4–12 beats) and long-term (α2, 13–64 beats) detrend fluctuation analysis slope and the correlation dimension (D2). The maximal rating of perceived exertion (RPE), during daily exercise, was assessed using the Borg 6–20 RPE scale.

**Results:** All subjects completed the HA exposure. The average age of included subjects was 31.4 ± 8.1 years. HA led to a significant fall in SpO_2_ and increase in heart rate, LLS and RPE. There were no significant changes in the ECG-derived respiratory rate or in any of the time domain measures of HRV during sleep. The only notable changes in frequency domain measures of HRV were an increase in LF and fall in HFnu power at the highest altitude. Conversely, SampEn, SD1/SD2 and D2 all fell, whereas α1 and α2 increased (*p* < 0.05). RPE inversely correlated with SD1/SD2 (*r* = -0.31; *p* = 0.002), SampEn (*r* = -0.22; *p* = 0.03), HFnu (*r* = -0.27; *p* = 0.007) and positively correlated with LF (*r* = 0.24; *p* = 0.02), LF/HF (*r* = 0.24; *p* = 0.02), α1 (*r* = 0.32; *p* = 0.002) and α2 (*r* = 0.21; *p* = 0.04). AMS occurred in 7/16 subjects (43.8%) and was very mild in 85.7% of cases. HRV failed to predict AMS.

**Conclusion:** Non-linear HRV is more sensitive to the effects of HA than time and frequency domain indices. HA leads to a compensatory decrease in nocturnal HRV and complexity, which is influenced by the RPE measured at the end of the previous day. HRV failed to predict AMS development.

## Introduction

High altitude (HA) exposure leads to a number of well recognized physiological responses under hypobaric hypoxia (West, 2006). These include increases in resting minute ventilation and pulmonary artery systolic pressure (West, 2006). Resting cardiac output increases which is principally driven by a rise in resting heart with little change in stroke volume (Boos et al., 2016b).

The influence of HA on the changes in cardiac inter-beat intervals (IBI), known as heart rate variability (HRV), has been an area of significant recent research interest (Huang et al., 2010; Karinen et al., 2012; Boos et al., 2016a, 2017b; Mellor et al., 2017). This attention relates, in part, to the fact many of the factors that are known to affect HRV (e.g., fatigue, stress, insomnia, hypoxia, and cold) are predominant at HA (West, 2006; Kemp et al., 2012; Kiviniemi et al., 2014). The improved portability and reduced cost of HRV recording equipment has helped to create new research opportunities at HA, that were previously untenable. Cardiac patch monitoring represents a significant advance in this regard. Patch monitors can non-invasively and accurately measure the cardiac IBIs, whilst negating the need for intrusive chest straps or electrocardiogram cables that are prone to interference and detachment. Despite these advantages, their utility to assess HRV at HA has not been examined.

There is evidence to suggest that HA exposure is associated with significant changes in HRV compared with sea-level/low altitude (Huang et al., 2010; Karinen et al., 2012; Boos et al., 2016a, 2017b; Mellor et al., 2017). However, the majority of the published data relate to short-term HRV recordings (1–5 min) obtained conducted in hypoxic chambers during ‘simulated’ rather than genuine terrestrial HA (Vigo et al., 2010; Prabhakaran and Tripathi, 2011; Mairer et al., 2013; Zhang et al., 2014). The hypoxic period examined has been generally brief (minutes to <8 h) with a tendency to assess at a single HA (Saito et al., 2005; Sutherland et al., 2017) leading to an under appreciation of the influence of acclimatization on HRV. Furthermore, despite the known influence of sleep on autonomic function, there has been a distinct lack of research into nocturnal HRV at HA (Chouchou and Desseilles, 2014; Taralov et al., 2015).

Despite these research limitations there is some, albeit limited data, supporting a potential link between changes in HRV at HA and acute mountain sickness (AMS) development (Huang et al., 2010; Karinen et al., 2012; Boos et al., 2017b; Sutherland et al., 2017). However, there are marked inconsistencies in the published results. This may relate to the heterogeneity, in the methods used to assess HRV, the HA environment (terrestrial versus simulated; severity and duration of hypoxia), exercise intensity and in the populations studied (Mellor et al., 2014; Boos et al., 2016a).

There is an increasing appreciation that traditional time and frequency domain measures of HRV that have dominated the literature, provide an incomplete representation of the complexity of IBI variability and autonomic balance (Sassi et al., 2015). Consequently, a number of non-linear measures of HRV have emerged that provide incremental and prognostic data (Sassi et al., 2015). To date these parameters have barely been examined at terrestrial HA (Di Rienzo et al., 2010).

In this study we aimed to assess, for the first time, the use of a cardiac patch monitor to assess both linear and non-linear measures of HRV at terrestrial HA.

## Materials and Methods

### Subjects

**FIGURE 1:**
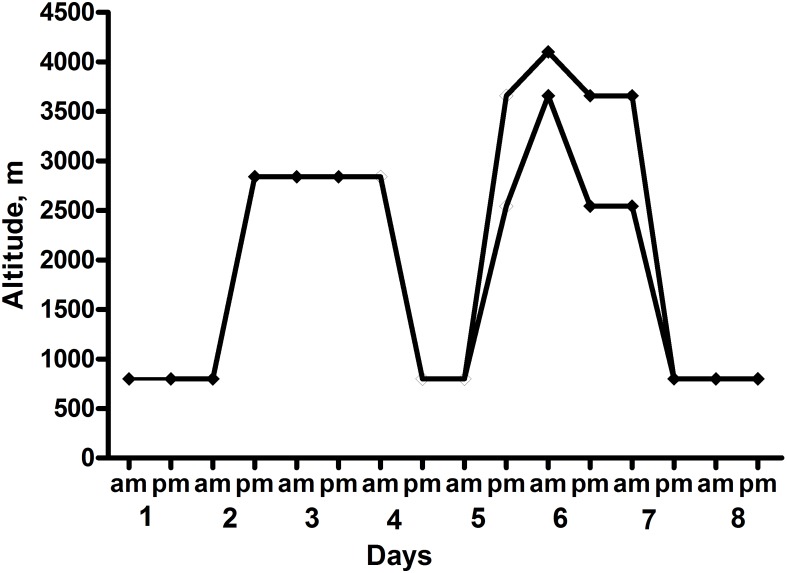
Ascent Profile of the subjects. Heart rate variability (HRV) recordings were taken at 0200–0300 each night.

Sixteen healthy British military servicemen, undergoing military training in the Bernese Alps, were studied (the ascent profile is shown in **Figure 1**). All participants arrived by road to their 800 m basecamp, where they spent their first night. Thereafter, their second and third nights were at 2840 m (accessed by road to 1200 m then on foot over 4 h carrying a weight of 15 kg). During their days at 2840 m they underwent training serials on a nearby glacier. Their fourth night was spent back at the 800 m basecamp. Following this they were split into two equal groups of eight participants with one group of eight spending their fifth and sixth nights in huts at 3658 m and the other at 2543 m. Those based at 3658 m underwent a daytime HA acclimatization climb to 4100 m, whereas those at 2543 m climbed to 3583 m. Both groups descended to their huts to sleep. All subjects spent their seventh and eighth nights back at basecamp (800 m) where they stayed till the end of the data collection (day 9). The subjects slept in sleeping bags in tents at 800 m and in beds with sleeping bags in huts at the higher altitudes.

### Physiological and Physical Assessments

Assessment of SpO_2_ and HA related symptoms were measured, during seated rest, in the early morning at each altitude. HA-related symptoms were recorded using the Lake Louise Scoring (LLS) system (Roach et al., 1993). This allocates a symptom score ranging from 0 (none) to 3 (severe) to the following five symptoms: difficulty sleeping, gastrointestinal symptoms, fatigue/weakness, dizziness/lightheadedness, and headache. A LLS of ≥3 in the presence of headache and a recent altitude gain was used to define AMS as previously described (Roach et al., 1993; Boos et al., 2016a). Mild AMS was defined as the presence of AMS and a LLS of 3–4 and severe when the LLS was ≥5 (Roach et al., 1993; Boos et al., 2016a). The rating of perceived exertion (RPE) during the day (sessional RPE) was assessed at the end of each day, using a 6–20 scale as previously described (Borg, 1970; Foster et al., 2001; Mellor et al., 2014).

### Assessment of Heart Rate Variability

**FIGURE 2:**
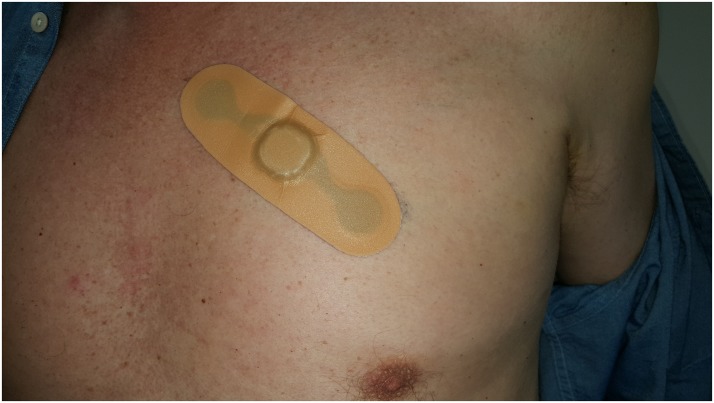
Illustration of the Health strip Cardiac Patch Monitor.

Continuous cardiac IBIs for each subject were recorded using a lightweight (6 grams) disposable 120 × 42 × 5 mm adhesive patch monitor (Health strip, LumiraDx; Quinlan et al., 2015) that was provided for this study free of charge. It was made available for investigational use in this study and is currently awaiting a CE mark. The Health strip contains two internal hydrogel electrodes (**Figure 2**). The patches were placed following simple skin preparation (shaving and an alcohol wipe). The subjects wore their patches continuously throughout the study period and the patches were replaced as necessary. The patches were placed over the second to the fourth intercostal space at angle of 45 degrees (toward the cardiac apex). The data was stored on the patch monitor prior to daily data Bluetooth transfer to an iPhone 6s smartphone. This coded cardiac data were then securely transferred using Wi-Fi for later HRV analysis.

Heart rate variability was assessed over a 1 h nocturnal cardiac recording period from 0200 to 0300 h during sleep each of the eight consecutive nights. The Health strip also records physical activity and body position (upright, supine on back, lying on side) by a movement sensor, which supports the confirmation of genuine sleep, which was documented as an activity percentage/minute. A 36 s full disclosure single lead ECG (Sampling frequency 250 Hz) was additionally recorded over the middle 36 s (0230 h) of each 1 h.

Heart rate variability analysis of the exported nocturnal 1 h IBI data from the Health strip were performed on desktop computer using dedicated HRV software (Kubios^®^ Premium ver. 3.0.2)^1^ as previously described (Tarvainen et al., 2014). Prior to HRV computation all IBI data were visually inspected for correctness and then underwent automatic artifact correction. The default sample length was set to 3600 s (1 h) and over the number of IBIs generated in this time frame.

Time and frequency domain measures of HRV were calculated according to the HRV Task Force Guidelines (Task Force of the European Society of Cardiology and the North American Society of Pacing Electrophysiology, 1996) The following established time domain measures of HRV were assed: SDNN (standard deviation of the NN intervals), RMSSD (root mean square of successive differences), pNN50% (the number of NN intervals that differ by >50 ms divided by the total number of NN intervals). Prior to calculation of the spectral HRV parameters, the Kubios default smoothness priors detrending was employed (Lambda, λ value = 500) as previously described (Tarvainen et al., 2014; Gąsior et al., 2016). The IBIs were transformed to evenly sampled time series with 4-Hz interpolation resampling rate. The detrended and interpolated IBIs were used for the frequency-domain HRV analysis. HRV spectra were calculated by using the fast Fourier transform (FFT) with Welch’s periodogram method (50% overlap window and 300 s window width) as previously described (Gąsior et al., 2016). We reported the low-frequency (LF) (0.04–0.15 Hz) and HF (high-frequency) power (0.15–0.40 Hz) and the LF/HF ratio as previously described (Task Force of the European Society of Cardiology and the North American Society of Pacing Electrophysiology, 1996). Due to skewed distributions, LF and HF power were transformed by natural logarithms (ln). In order obtain greater insight into the relative HF power it was also reported in normalized units HF (HFnu) which was calculated as HF/(LF + HF) (Task Force of the European Society of Cardiology and the North American Society of Pacing Electrophysiology, 1996). Non-linear HRV assessment were examined as previously described (Sassi et al., 2015) using Poincaré plots and the derived ratio of the standard deviation (SD) of long term (SD2) to short term (SD1) variability known as SD1/SD2 ratio, Sample entropy (SampEn), the short (α1, 4–12 beats) and long term detrend fluctuation analysis (DFA) slopes (α2, 13–64 beats) and the correlation dimension (D2) (Sassi et al., 2015). The Poincaré plot is a scatterplot in which current IBIs are plotted as a function of previous interval. SD1 represents the SD of short-term HRV and SD2 (or major axis) the continuous longer term IBIs (Task Force of the European Society of Cardiology and the North American Society of Pacing Electrophysiology, 1996). The SD1/SD2 ratio measures the unpredictability of the RR time series (Shaffer and Ginsberg, 2017). SampEn is a measure of the regularity and fluctuation of a time series with lower values representing less complexity and greater self-similarity in a time series (Richman and Moorman, 2000). DFA detects the simple correlations between successive RRIs over differing time scales with α1 reflecting the slope over shorter fluctuations and α2 over longer time periods (Shaffer and Ginsberg, 2017). D2 is an estimation of the number of independent variables necessary to describe a systems behavior with a higher value representing greater complexity (Sassi et al., 2015).

Calculation of the respiratory rate was obtained by ECG-derived respiration (EDR) software within the HRV analysis package as previously described, using the 36 s Health strip ECG recording (Babaeizadeh et al., 2011).

### Statistical Analysis and Sample Size Calculation

Data were analyzed using GraphPad InStat version 3.05 and with all graphical figures presented using GraphPad Version 3.10 (GraphPad Software, San Diego, CA, United States) ^2^. Data inspection and the Kolmogorov–Smirnov test was undertaken to assess normality of all continuous data. Results are presented as mean ± SD for all data. Comparison of continuous data between altitudes was performed using a one-way ANOVA with Tukey posttest and with Kruskal–Wallis test with Dunn-Posttest for parametric and non-parametric data, respectively. Correlations of continuous data were assessed using the Pearson and Spearman rank correlation coefficients (r) and 95% confidence interval, for parametric and non-parametric data, respectively. A two-sided *p*-value of < 0.05 was considered as significant for all analyses.

In a previous, yet recent study, we had observed a significant 11% (7.9 ms) change in the RMSSD-derived HRV score between baseline sea level and 3619 m in a cohort of 12 persons (Boos et al., 2016a). Zhang et al. (2015) recently observed a significant fall in SampEn in eight healthy male subjects, following short term exposure to simulated HA from sea level to 3600 m. Based on this later data, we calculated that a sample size of at least 14 subjects would have sufficient power to examine for differences in RMSSD and a ≥80% to detect a difference in mean SampEn of ≥0.18 at a significance level (alpha) of 0.05 (two-tailed) (GraphPad Statmate).

### Ethics Statement

All participation was entirely voluntary and all subjects underwent detailed written informed consent >24 h after being sent a participant information sheet for the study. This study was approved by the Ministry of Defence Research and Medical Ethics Committee (MODREC) and was conducted according to the standards of the Declaration of Helsinki.

## Results

The average age of included subjects was 31.4 ± 8.1 years. They had mean height of 179.8 ± 5.0 cm, weight of 84.6 ± 11.0 kg and body mass index of 26.1 ± 2.7 kg/m^2^. All of the participants were non-smokers and were not on any regular medication.

Compared with baseline, ascent to HA ≥2543 m was associated with a significant fall in SpO_2,_ higher RPE scores, and an increase in average heart rate, LLS and in the average sleep score component of the LLS (**Table 1**).

**Table 1:** Changes in physiological measurements, Lake Louise scores and rating of perceived exertion (RPE).

Altitude	800 m (1)	2840 m	800 m (2)	2543–3658 m	800 m (3)	*P*-value
SpO_2_, %	96.8 ± 1.2	92.2 ± 2.4	96.1 ± 2.0	90.0 ± 3.3	95.9 ± 3.0	<0.0001^acef^
Mean heart rate, minute^-1^	53.2 ± 7.3	68.8 ± 14.9	68.9 ± 17.9	65.0 ± 13.4	60.8 ± 9.3	0.001^abc^
ECG-derived Respiratory rate	16.7 ± 3.9	17.3 ± 2.0	16.8 ± 3.6	16.9 ± 3.4	16.9 ± 4.6	0.90
Lake Louise total scores	0.2 ± 0.6	1.0 ± 1.1	0.5 ± 1.2	1.4 ± 1.5	0.4 ± 0.70	<0.0001^acf^
Lake Louise sleep scores	0.0 ± 0.2	0.6 ± 0.8	0.0	0.3 ± 0.7	0.1 ± 0.5	<0.0001^ae^
RPE score	10.8 ± 2.8	11.40 ± 2.6	10.2 ± 3.5	12.4 ± 2.5	10.4 ± 1.9	0.02^cef^

*SpO_*2*_, capillary oxygen saturation; RPE, rating of perceived exertion (Borg 14–20 score). Significant post-test differences vs. baseline 800 m (1): a 2840 m; b 800 m (2); c 2543–3658 m; d 800 m (3); e 800 m (2) vs. 2840 m; f 800 m (2) vs. 2543–3658 m*.

The mean number of patches used per subject over the study was 1.94 ± 0.25 (range 1–2), with all, but one subject, requiring two patches. The recorded cardiac IBI data were good quality with an artifact rate of < 3% at all altitudes studied, but was significantly higher at higher altitude at ≥2543 m versus baseline 800 m (**Table 1**). There were no significant overall changes in any of the time domain measures of HRV (**Table 2**). The only notable change in frequency domain measures of HRV was an increase in LF power and fall in HFnu at the highest altitude (**Table 2**). There were significant changes in all of the non-linear measures of HRV at HA: SampEn, SD1/SD2 and D2 fell, whereas α1 and α2 increased (*P* < 0.05) (**Table 3**).

**Table 2:** Comparative time and frequency domain heart rate variability (HRV) parameters at high altitude (HA).

Altitude	800 m (1)	2840 m	800 m (2)	2543–3658 m	800 m (3)	*P*-value
Artifact %	1.1 ± 1.1	2.4 ± 1.8	1.6 ± 1.0	2.6 ± 1.7	1.1 ± 0.7	0.001^acg^
LnSDNN	4.2 ± 0.4	4.3 ± 0.6	4.2 ± 0.7	4.5 ± 0.40	4.0 ± 0.8	0.09
LnRMSSD	4.4 ± 0.5	4.2 ± 0.8	4.2 ± 0.8	4.40 ± 0.60	4.0 ± 0.9	0.45
pNNI%	39.4 ± 18.9	28.9 ± 23.3	21.8 ± 25.1	37.7 ± 18.4	25.4 ± 22.7	0.35
Triangular index	15.1 ± 3.70	15.1 ± 8.70	16.6 ± 13.3	19.6 ± 11.2	14.2 ± 9.1	0.09
LnLF	7.6 ± 0.6	7.6 ± 1.0	7.5 ± 1.3	8.4 ± 0.8	7.0 ± 1.5	0.004^eg^
LnHF	7.3 ± 1.0	7.1 ± 1.6	7.2 ± 1.6	7.5 ± 1.2	6.8 ± 1.6	0.59
HFnu,%	40.5 ± 15.1	36.4 ± 17.4	43.3 ± 16.3	31.9 ± 16.0	43.3 ± 15.7	0.04^fg^
LF/HF ratio	1.7 ± 1.0	2.8 ± 2.6	1.7 ± 1.2	2.9 ± 2.0	1.6 ± 01.1	0.09

*SDNN, standard deviation of normal-to-normal; RMSSD, root mean square of successive differences; pNNI%, Percentage of successive RRs >50 ms; LF, Low frequency; HF, high frequency; Significant post-test differences: vs. baseline 800 m (1), a 2840 m, b 800 m (2), c 2543–3658 m, d 800 m (3); e 800 m (2) vs. 2840 m; f 800 m (2) vs. 2543–3658 m; g 800 m (3) vs. 2543–3658 m*.

**Table 3:** Effect of HA on Non-linear measures of HRV.

Altitude	800 m (1)	2840 m	800 m (2)	2543–3658 m	800 m (3)	*P*-value
SD1/SD2	0.6 ± 0.1	0.5 ± 0.2	0.6 ± 0.2	0.5 ± 0.2	0.6 ± 0.2	0.02^cf^
Sample entropy	1.6 ± 0.2	1.4 ± 0.3	1.6 ± 0.3	1.4 ± 0.2	1.7 ± 0.2	0.0004^ace^
DFA α1	1.0 ± 0.2	1.2 ± 0.3	1.0 ± 0.3	1.1 ± 0.2	0.9 ± 0.2	0.006^acef^
DFA α2	0.4 ± 0.10	0.5 ± 0.10	0.4 ± 0.1	0.4 ± 0.1	0.4 ± 0.2	0.03^ac^
D2	3.4 ± 1.4	2.1 ± 1.3	2.3 ± 1.5	2.9 ± 1.3	2.1 ± 1.6	0.03^ad^

*SD, standard deviation; DFA, detrend fluctuation analysis; D2, Correlation Dimension. Significant post-test differences vs. baseline 800 m (1): a 2840 m; b 800 m (2); c 2543–3658 m; d 800 m (3); e 800 m (2) vs. 2840 m; f 800 m (2) vs. 2543–3658 m*.

Rating of perceived exertion inversely correlated with HFnu, SD1/SD2 and SampEn and positively correlated with LF, LF/HF, α1, and α2, but not the other HRV indices (**Table 4**). Among the non-linear indices only SD1/SD2 inversely correlated with α1 (*r* = -0.87; -0.91 to -0.81: *p* < 0.0001), α2 (*r* = 0.28; -0.44 to -0.10: *p* = 0.003). SampEn inversely correlated with α2 (*r* = -0.27; -0.44 to -0.1: *p* = 0.003) and D2 inversely with α2 (*r* = -0.35; -0.51 to -0.17: *p* = 0.0001).

**Table 4:** Correlation between RPE and HRV measures

HRV parameter	Correlation coefficient	95% Confidence interval	*P*-value
HFnu	-0.27	-0.45 to -0.07	0.007
LF	0.24	0.04 to 0.44	0.02
LF/HF	0.24	0.04 to 0.42	0.02
SD1/SD2	-0.31	-0.49 to -0.10	0.002
Sample entropy	-0.22	-0.40 to 0.02	0.03
α1	0.32	0.11 to 0.49	0.002
α2	0.21	0.1 to 0.40	0.04

*LF, Low frequency; HF, high frequency; SD, standard deviation*.

Seven out of the sixteen subjects (43.8%) suffered with AMS. These were all self-limiting and in all but one were mild episodes (LLS score 3–4). HRV scores failed to predict AMS.

## Discussion

This is the first study to assess the utility of a cardiac patch monitor to assess non-linear measures of HRV at HA. We found that non-linear HRV was more sensitive to the effects of HA than traditional time and frequency domain HRV measurements. HA led to a significant fall in SD1/SD2, D2, and SampEn and an increase in α1 and α2. We observed a significant relationship between nocturnal HRV and RPE measured at the end of the previous day. HRV measures failed to predict the development of AMS.

We chose to examine the effects of HA on several non-linear HRV parameters, given the paucity of data at HA and their potential advantages over established time and frequency domain HRV parameters. Their advantages include its lower sensitivity to the presence of cardiac ectopy, artifacts and to the recording period, which is of greater relevance at HA (Voss et al., 2008). The non-linear HRV parameters examined in this study were the SD1/SD2 ratio obtained from Poincaré plots, SampEn, α1, and α2 and D2. We found that SampEn was significantly lower at HA at both 2840 and 2543–3658 m compared with baseline. Conversely the α1 and α2 increased from baseline to 2480 and 2543–3658 m, with a return to near baseline levels at 800 m. D2 and SD1/SD2 values were also significantly lower at HA versus 800 m. These findings suggest that HA to ≥2543 m leads to a compensatory change in autonomic balance with increased regularity (lower HRV) and lower complexity and chaos in the cardiac IBI signal (Shaffer and Ginsberg, 2017).

Our data is largely consisted with that obtained from several recent acute hypoxia studies. In their study of eight healthy men exposed to acute normobaric hypoxia (equivalent to 3600 m) for 10 min, Zhang et al. (2015) also reported a fall in SampEn, measured over a 1 min recording period. Their observed values of α1, which was also studied, were similar to our current study and very close to 1.0, but did not change significantly. In another study of ten healthy men, supine HRV was measured during intermittent periods of acute normobaric hypoxia (simulated HA; FIO_2_ down to 9.8%) (Giles et al., 2016). Again, a significant fall in SampEn was observed, but on this occasion it was associated with a significant increase in α1, which is consistent with our data. Due to the brevity of the HRV recording period in their study, α2 and other non-linear HRV measures were not examined.

In this study we assessed HRV during sleep at HA. Our interest in specifically examining this period was stimulated by a number of factors. Firstly, it has been well shown that sleep and its stages are associated with marked variability in autonomic modulation of cardiac activity that is typified higher parasympathetic tone during normal non-rapid eye movement sleep (REMS) and a shift toward sympathetic predominance during normal REMS (Tobaldini et al., 2013; Chouchou and Desseilles, 2014). Secondly, ventilation, which can have a significant influence on HRV, is affected by HA (Task Force of the European Society of Cardiology and the North American Society of Pacing Electrophysiology, 1996). Alterations in breathing patterns and even periodic breathing (PB) are a well-established phenomenon at HA (Burgess and Ainslie, 2016). PB represents an abnormal ventilatory pattern in which apneas and hypopneas alternate with periods of hyperventilation (Burgess and Ainslie, 2016). The worsening hypobaric hypoxia at HA leads to compensatory hyperventilation until a point when the arterial PCO_2_ (PaCO_2_) falls below the threshold required to stimulate breathing leading to either hypopnea or even apnea, followed by the restoration of hyperventilation as the hypoxia worsens and the paCO_2_ resets (Burgess and Ainslie, 2016). This phenomenon is subject to marked individual variability, but is generally observed at >2000 m (Burgess and Ainslie, 2016). Unfortunately, we were not able to measure ventilation throughout the 1 h recording period, but did quantify the ECG derived respiratory rate during part of the HRV recording period. It is highly likely that there were cases of PB and the observed swings in the IBI raise this suspicion. Nevertheless, wary of the confounding effect of sleep stage and potentially PB on HRV we selected a 1-h HRV recording period in preference to a traditional 5-min recording to minimize this potential bias (Task Force of the European Society of Cardiology and the North American Society of Pacing Electrophysiology, 1996).

By using an adhesive cardiac patch monitor (and avoiding ECG cables and minimizing movement artifact) we were able to overcome the obvious challenges of accurately, yet non-intrusively measuring HRV during sleep at HA. However, poor sleep was still a significant contributor to the total LLS at each altitude in our study. The sleep score component of the LLS was significantly higher at 2840 m compared with 800 m, indicating perceptually worse sleep at higher altitude (**Table 1**). Whilst reduced HRV with insomnia is a widely accepted concept, it has not been well supported by empirical evidence (Dodds et al., 2016). Reduced sleep quality and insomnia are common at HA, but its effect on HRV has not been examined. Unfortunately, we were unable to assess the sleep stages during the HRV recording or the total sleep time prior to HRV recording. However, the subjects generally went to sleep before 2300 h each night and the activity sensor on the Health strip confirmed that subjects were supine and largely inactive during the HRV defined nocturnal HRV recording period.

The significant, yet modest, relationship between end of day RPE and nocturnal HRV is a novel finding at HA. Our data support the temporal effects of heavy exercise and exhaustion on HRV (Taralov et al., 2015). Higher RPE appeared to be associated with lower nocturnal HRV and greater LF/HF dominance.

This study has a number of additional strengths and limitations that should be acknowledged. The fact that we were we studied three separate terrestrial altitudes, yet included a far larger sample size than the majority of published acute hypoxic chamber studies are obvious strengths. The wide breadth of HRV parameters and physiological measurement examined, allowed for a comprehensive assessment of HRV at HA. Baseline HRV studies were performed at 800 m rather than sea level due to practical issues, which could have reduced the effect size. It was not possible to control the subjects sleeping position (e.g., prone or on side) and their sleeping conditions varied with altitude which may be potential confounders (Ryan et al., 2003). The altitude studied was modest and the majority of AMS cases were mild, hence we cannot be certain whether our findings would be reproducible at higher altitudes and with worsening AMS severity. The duration and intensity of exercise varied with altitude, which whilst being a relevant confounder, reflects the reality of a real world terrestrial HA venture. We were only able to absolutely confirm the presence of normal sinus rhythm at the time of the 36 sec ECG capture. Whilst this does not fully exclude the possibility of arrhythmias at other time points visualization of the IBI data coupled with the altitude and healthy population studied would strongly suggest against the presence of an undetected significant cardiac arrhythmia (Boos et al., 2017a).

## Conclusion

This study demonstrated that moderate terrestrial HA exposure leads to significant changes in resting nocturnal non-linear HRV that is typified by increased regularity and lower complexity and chaos of the cardiac inter-beat signal. These changes are influenced by the intensity of exercise over the previous day. Nocturnal HRV was not predictive of AMS.

## Author Contributions

CB, LS, and KB performed all the experiments. CB, AM, DW, TQ, and MS initiated the project. CB, TQ, and LS performed the data analysis. All the authors contributed to data paper writing.

## Conflict of Interest Statement

LumiraDx supplied the patches and supplied intellectual input but were not directly involved in the main statistical analysis of the data nor the HRV analysis. The authors declare that the research was conducted in the absence of any commercial or financial relationships that could be construed as a potential conflict of interest.

## Footnotes

1 http://www.kubios.com/

2 www.graphpad.com
